# The novel technique of drainage stenting using a tapered sheath dilator in endoscopic ultrasound‐guided biliary drainage

**DOI:** 10.1002/deo2.303

**Published:** 2023-10-22

**Authors:** Akihisa Kato, Michihiro Yoshida, Yasuki Hori, Kenta Kachi, Hidenori Sahashi, Tadashi Toyohara, Akihisa Adachi, Kayoko Kuno, Yusuke Kito, Hiromi Kataoka

**Affiliations:** ^1^ Department of Gastroenterology and Metabolism Nagoya City University Graduate School of Medical Sciences Aichi Japan

**Keywords:** biliary drainage, bile leakage, Endosheather, EUS‐BD, tapered sheath dilator

## Abstract

During endoscopic ultrasound‐guided biliary drainage (EUS‐BD), there is a risk for bile leakage until stent deployment, which can result in severe peritonitis, particularly when passing a drainage stent becomes challenging despite tract dilation. There is no established method or dedicated device to optimize EUS‐BD. Therefore, we have developed a novel stent deployment technique using the tapered sheath dilator. To address the safety and technical aspects of the EUS‐BD technique, we retrospectively analyzed 11 consecutive patients who underwent EUS‐BD using the tapered sheath dilator. The procedure involved the insertion of a guidewire, followed by mechanical dilation using the tapered sheath dilator. Subsequently, the inner catheter was removed and drainage stents (up to 6 Fr in diameter) were deployed through the outer sheath. We found a 100% technical success rate for tract dilation and stent deployment; moreover, all patients achieved clinical success. The median time for dilation was 40 s (range, 8–198), whereas the median time from dilation to stent deployment was 10 min (range, 6–19). Notably, no cases of bile leakage or peritonitis were observed. In conclusion, the use of the integrated device for tract dilation and stent delivery system might provide a safe and straightforward technique for drainage stenting during EUS‐BD.

## INTRODUCTION

Endoscopic ultrasound‐guided biliary drainage (EUS‐BD) is a highly effective treatment option for various conditions, including obstructive jaundice, cholangitis, and cholecystitis.^1^, ^2^ However, the deployment of a drainage stent through a needle tract poses a significant challenge, even after dilation. This prolonged interval between dilation and stent deployment can lead to bile leakage through the dilated tract, potentially resulting in infected biloma or severe peritonitis. Despite numerous attempts to address this issue, a standardized method has yet to be established.

Therefore, we have developed a novel stent deployment technique using an endoscopic tapered sheath (EndoSheather; Piolax, Inc.)^3^, ^4^; this device serves as a dilation tool and a delivery system, allowing for mechanical dilation of the needle tract and the smooth insertion of a drainage stent through its indwelling outer sheath, which effectively bridges the gap to the target space. This eliminates the need to remove the dilation device and can prevent bile leakage in EUS‐BD.

## PROCEDURE OR TECHNIQUE

### Procedure

**FIGURE 1:**
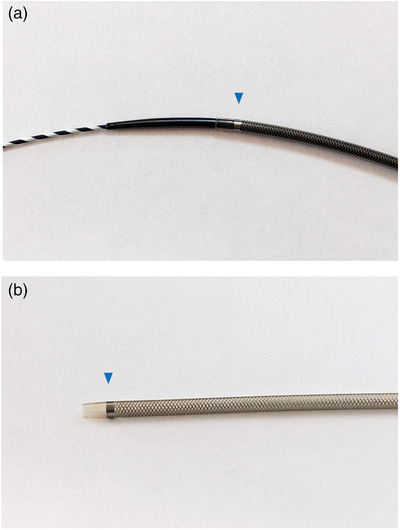
Appearance of the endoscopic tapered sheath (EndoSheather). (a) Minimal difference in caliber between the inner catheter and the outer sheath at the device tip. (b) The outer sheath with a mesh braided structure which provides optimal kink resistance. Presence of a radiopaque marker at the tip of the outer sheath (blue arrowhead). The internal and external diameters of the outer sheath are 6.2 Fr (2.06 mm) and 7.2 Fr (2.44 mm), respectively.

**FIGURE 2:**
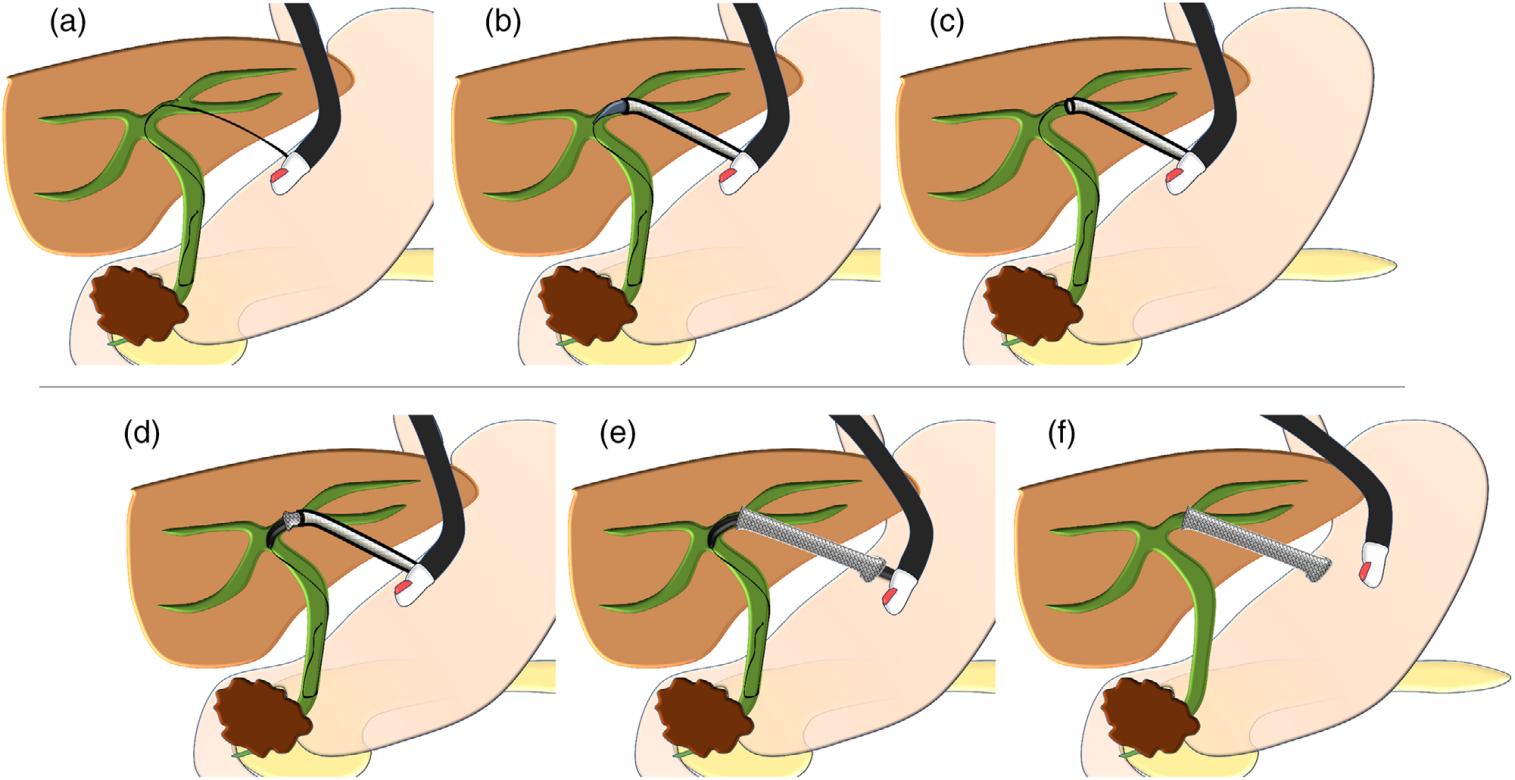
Illustrations demonstrating the stenting technique using the tapered sheath dilator (EndoSheather) in endoscopic ultrasound‐guided hepaticogastrostomy (EUS‐HGS). (a) Proper insertion of a guidewire. (b) Utilization of the tapered sheath dilator to mechanically dilate the needle tract. (c) Removal of the inner catheter, leaving the outer sheath in position within the bile duct. (d–f) Smooth insertion and deployment of a fully covered self‐expandable metal stent (up to 6 Fr in diameter) through the outer sheath.

**FIGURE 3:**
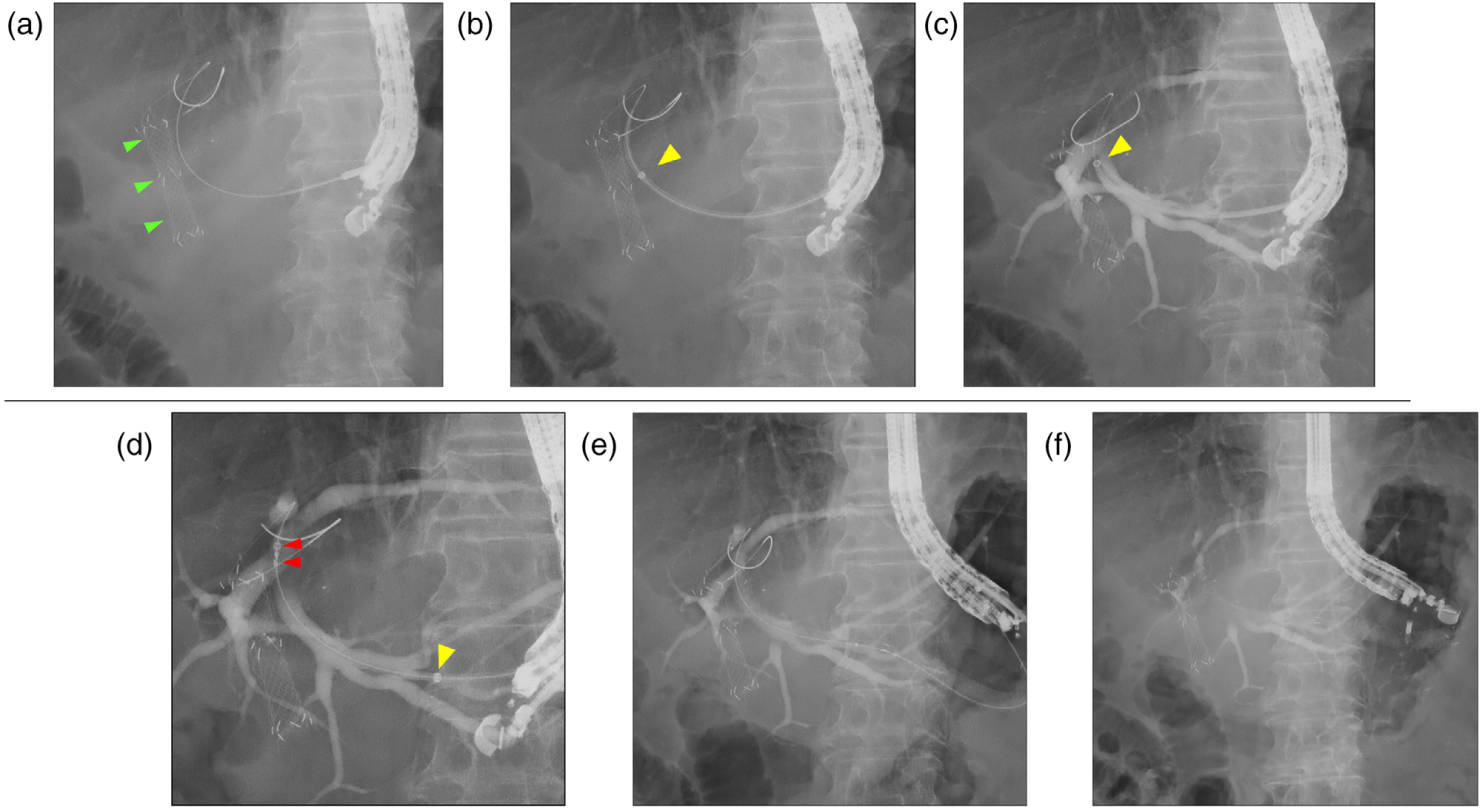
Fluoroscopic cholangiogram showing the stenting technique using the tapered sheath dilator (EndoSheather) in endoscopic ultrasound‐guided hepaticogastrostomy (EUS‐HGS). Green arrowheads indicate the deployed stent under endoscopic retrograde cholangiopancreatography. (a) Proper insertion of a guidewire. (b) Utilization of the tapered sheath dilator to mechanically dilate the needle tract. Yellow arrowhead indicates the radiopaque marker of the outer sheath. (c) Removal of the inner catheter, leaving the outer sheath in position within the bile duct. The outer sheath enabled aspiration of bile juice and injection of contrast medium. (d–f) Smooth insertion and deployment of a fully covered self‐expandable metal stent with a 5.9 Fr delivery system. Red arrowheads show the markers of the metal stent.

EUS‐BD was performed in all patients in a prone position using a linear echoendoscope (GF‐UCT260; Olympus Medical Systems) and CO_2_ insufflation. The bile duct or gallbladder was punctured under EUS guidance using a 19‐gauge needle and a small amount of contrast medium was injected to confirm accurate placement. A 0.025‐inch guidewire was inserted, followed by the mechanical dilation of the needle tract using a novel guide sheath with a tapered tip of the inner catheter (EndoSheather; Figure 1). Subsequently, the inner catheter was removed, whereas the outer sheath remained in place inside the bile duct or gallbladder. The outer sheath with a Y‐connector attachment facilitated effective aspiration of bile juice and injection of contrast medium to ensure accurate stent positioning. Finally, drainage stents (up to 6 Fr in diameter) were selected based on the specific requirements of each case and deployed through the outer sheath (Figures 2 and 3, and Video S1). The stents used in our study included fully covered self‐expandable metal stents with a 5.9‐Fr (8 mm × 8 cm/12 cm; HANAROSTENT Biliary Full Cover Benefit; Boston Scientific Co.), an uncovered self‐expandable metal stents with a 5.7‐Fr (8 mm × 6 cm; BileRush Selective; Piolax) and 5.4‐Fr (8 mm × 6 cm; Zeo stent V; ZEON Medical Inc.), and a plastic stent with a 6‐Fr (4/7/10 cm; Zimmon double pig‐tail stent; Cook Medical) delivery systems.

### Patients and methods

We retrospectively analyzed 11 consecutive patients who underwent EUS‐BD using a tapered sheath dilator at Nagoya City University Hospital between August 2021 and March 2023. The indications for the procedure were determined at the discretion of the endoscopists. The review board of the Nagoya City University Graduate School of Medical Sciences (approval no. 60–22–0052) approved the study protocol; informed consent was obtained from all patients.

The primary endpoint was to determine the incidence rate of early adverse events, including bile leakage and peritonitis, within 2 weeks. Especially, bile leakage was based on the findings around the puncture site of computed tomography on the day following the EUS‐BD. In addition to this finding, a diagnosis of peritonitis can be made with fever (>38°C), abdominal pain with peritoneal irritation signs (not caused by pancreatitis or perforation), or an elevated inflammatory response (an increase from baseline). Adverse events were classified according to the American Society for Gastrointestinal Endoscopy lexicon.^5^ Besides, we assessed the technical feasibility and efficacy of the procedure as secondary endpoints. Technical success was defined as successful tract dilation and subsequent stent deployment. Clinical success for cholestasis was determined by a reduction > 50% or normalization of the plasma bilirubin within 14 days, following the Tokyo criteria 2014,^6^ and for cholecystitis, it was defined as the resolution of clinical symptoms and laboratory findings associated with cholecystitis. Furthermore, we measured the duration of dilation, the time interval between dilation and stent deployment, and the overall procedure time, defined as the time from the first puncture to stent deployment.

## RESULTS

Table 1 presents the baseline clinical characteristics of all patients. The study included four male and seven female patients, with a median age of 73 (range, 52–97) years. Among the patients, seven had malignancies, such as pancreatic cancer, whereas four had benign conditions, such as cholecystitis. EUS‐BD was an alternative management approach to address challenging situations where conventional transpapillary techniques were unacceptable. The drainage targets were the bile duct in eight (72.7%) and the gallbladder in three (27.2%) patients. The objectives of drainage were to control jaundice in six (54.5%) and infection in six (54.5%) patients.

**TABLE 1:** Clinical characteristics.

Characteristic	Patients (*n* = 11)
Gender (male/female)	4/7
Age (years), median (range)	73 (52–97)
Primary biliary disease	
Malignant (*n* = 7)	
Pancreatic cancer	4
Bile tract cancer	2
Gastric cancer	1
Benign (*n* = 4)	
Cholecystitis	3
Anastomosis stricture	1
Target to drainage	
Bile duct	8
Gallbladder	3
Purpose of drainage	
Improvement of jaundice	6^*^
Infection control	6^*^
Presence of cholangitis before drainage	4
Total bilirubin (mg/dL), median (range)	1.3 (0.5–13.0)

* One case is duplicated.

Table 2 presents the details of each EUS‐BD. Among the patients, EUS‐guided hepaticogastrostomy (EUS‐HGS) was performed in four, EUS‐guided antegrade stenting (EUS‐AS) in three, EUS‐guided gallbladder drainage (EUS‐GBD) in three, and EUS‐guided hepaticoduodenostomy (EUS‐HDS) in one. Regarding tract dilation, the technical success rate was 100% (11/11), with a median dilation time of 40 s (range, 8–198). Subsequent stent placement was successful in all patients (11/11), with a median time from dilation to stent deployment of 10 min (range, 6–19). The median procedure time was 23 min (range, 14–95). Furthermore, clinical success was achieved in all patients (11/11) who presented with jaundice and cholangitis.

**TABLE 2:** Details of each EUS‐guided biliary drainage procedure.

#	Disease	Procedure	Technical outcome	Puncture site	Diameter of the bile duct (mm)	Length of the hepatic parenchyma (mm)^†^	Time for dilation (s)
1	Pancreatic cancer	EUS‐HGS	Success	B3	5.0	20	40
2	Hilar cholangiocarcinoma	EUS‐HGS	Success	B3	4.0	35	20
3	Pancreatic cancer	EUS‐HGS	Success	B3	4.5	21	56
4	Anastomosis stricture	EUS‐HGS	Success	B3	4.0	14	8
5	Hilar cholangiocarcinoma	EUS‐HDS	Success	B6	5.1	13	11
6	Pancreatic cancer	EUS‐AS	Success	B3	4.7	20	110
7	Gastric cancer	EUS‐AS	Success	B3	2.0	17	12
8	Pancreatic cancer	EUS‐AS	Success	B3	4.0	25	9
9	Acute cholecystitis	EUS‐GBD	Success	Gallbladder	‐	‐	141
10	Acute cholecystitis	EUS‐GBD	Success	Gallbladder	‐	‐	169
11	Acute cholecystitis	EUS‐GBD	Success	Gallbladder	‐	‐	198

#	Clinical outcome	Stents used (diameter × length)	Time from dilation to stent deployment (min)	Any symptoms after the procedure	Early adverse event
1	Success	8 mm × 120 mm FCSEMS	10	‐	‐
2	Success	8 mm × 120 mm FCSEMS	10	‐	‐
3	Success	8 mm × 120 mm FCSEMS	10	Fever	Cholangitis
4	Success	8 mm × 120 mm FCSEMS	7	‐	‐
5	Success	6 Fr × 100 mm DPPS	6	‐	‐
6	Success	8 mm × 60 mm UCSEMS (/8 mm 12 cm FCSEMS)^⁑^	10	‐	‐
7	Success	8 mm × 60 mm UCSEMS (/7 Fr plastic stent 14 cm)^⁑^	7	‐	‐
8	Success	8 mm × 80 mm FCSEMS (/8 mm 10 cm FCSEMS)^⁑^	8	‐	‐
9	Success	8 mm × 80 mm FCSEMS	12	‐	‐
10	Success	8 mm × 80 mm FCSEMS	10	‐	‐
11	Success	8 mm × 120 mm FCSEMS	19	‐	‐

Abbreviations: DPPS, double pigtail plastic stent; EUS‐AS, EUS‐guided antegrade stenting; EUS‐GBD, EUS‐guided gallbladder drainage; EUS‐HDS, EUS‐guided hepaticoduodenostomy; EUS‐HGS, EUS‐guided hepaticogastrostomy; FCSEMS, fully‐covered self‐expanded metallic stent; UCSEMS, uncovered self‐expanded metallic stent.

^†^ Length of the hepatic parenchyma was measured from the bile duct to the periphery of the liver on the EUS image.

^⁑^ (/), the stent placed for the dilated needle tract in EUS‐guided antegrade stenting.

Regarding the primary endpoint of early adverse events, non‐occlusion moderate cholangitis, necessitating 5 days of conservative treatment, occurred in one patient (9.1%) on the day after the procedure. Meanwhile, no cases of bile leakage or peritonitis were observed.

## DISCUSSION

EUS‐BD has gained increasing importance in cases where conventional endoscopic retrograde cholangiopancreatography has been unsuccessful, particularly when access to the papilla is difficult due to duodenal obstruction or surgically altered anatomy.^7^, ^8^ Over the past decade, significant technical advancements and a growing body of clinical experience have been observed in the field of EUS‐BD. However, several important factors need to be addressed in terms of safety and technical aspects to optimize EUS‐BD.

The EUS‐BD involves puncture, guidewire placement, dilation of the needle tract, and stent deployment. Each of these steps is critical to the overall success of the procedure; particular attention should be given to the dilation of the needle tract, and stent deployment in terms of safety. Following the dilation, there is a risk for bile juice leakage until stent deployment, which can lead to infected biloma or severe peritonitis.

Several studies have investigated strategies to prevent bile leakage during EUS‐BD. Kawakubo et al.^9^ demonstrated that the use of a covered metal stent is more effective than plastic stents in preventing bile leakage after EUS‐HGS; however, this strategy alone does not reduce bile leakage during the EUS‐BD. Ishiwatari et al.^10^ reported that aspirating >10 mL bile before stent deployment during EUS‐HGS can reduce the risk for adverse events, including bile leakage and cholangitis. Similarly, Yamamoto et al.^11^ suggested that maintaining a distance of ≥ 2.5 cm between the intrahepatic bile duct and the liver periphery during EUS‐HGS can reduce the incidence of bile peritonitis. However, regardless of the method used, a certain degree of bile leakage can still occur following tract dilation.

The development of a safer and more reliable modality for EUS‐BD is of utmost importance to promote its widespread adoption, particularly among novice interventional endoscopists. Tract dilation techniques vary across different facilities, including the use of an ultra‐tapered mechanical dilator, balloon catheter, or electrocautery dilator.^12^ Honjo et al. reported that the use of mechanical dilators in EUS‐HGS has resulted in fewer adverse events and a high technical success rate.^13^ However, despite tract dilation, passing a drainage stent, whether metal or plastic, through a needle tract can be challenging; this can be attributed to insertional axis deviation or inadequate tract dilation. Consequently, the procedure time may be prolonged and additional over‐dilation using an alternative device may be needed, resulting in an increased risk for adverse events.

Park et al.^14^ emphasized the effectiveness of the one‐step metal stent placement technique using a tapered metal tip; this technique has been recommended to address safety and technical concerns. Although this technique demonstrated a one‐step technical success rate of 88% (14/16) without the need for additional dilation, tract dilation was still required in two patients. Therefore, we developed an improved stenting technique using an integrated device for tract dilation and a stent delivery system.

The novel endoscopic tapered sheath (EndoSheather) was initially designed to acquire biopsy samples to diagnose biliary strictures.^4^ This device facilitates the smooth insertion of biopsy forceps to the targeted lesion during endoscopic retrograde cholangiopancreatography. It also features a tapered tip on the inner catheter and a closely fitting outer sheath, which ensures excellent pushability and serves as a dilation device. In addition, the outer sheath with a mesh braided structure provides optimal kink resistance, which makes it suitable for use as a delivery system. Therefore, the utilization of the tapered sheath for dilation and stent delivery during EUS‐BD can effectively address safety and technical concerns. Our study demonstrated a 100% technical success rate for tract dilation and stent deployment; moreover, all patients achieved clinical success. Notably, no cases of bile leakage or peritonitis were observed.

Our study had several limitations. First, it was a retrospective study conducted at a single center. Second, it had a small sample size. Third, there are areas for improvement in the EUS‐BD technique, particularly regarding the selection of stents. Future advancements in stent devices are expected to enhance the applicability of this technique. Despite these limitations, our study provides valuable insights into the safe and effective stenting in the EUS‐BD. However, a prospective randomized controlled trial involving a larger sample size is warranted to further validate our findings.

In conclusion, the use of the integrated device for tract dilation and stent delivery might provide a safe and straightforward technique for drainage stenting during EUS‐BD. Our innovative approach could contribute to the development of optimal methods for EUS‐BD.

## CONFLICT OF INTEREST STATEMENT

None.

## Supporting information

Video S1. Demonstration of a novel stenting technique using a tapered sheath dilator for tract dilation and seamless insertion of a covered metal stent through the outer sheath, effectively preventing bile leakage during endoscopic ultrasound‐guided hepaticogastrostomy (EUS‐HGS).
